# Enhanced pyruvate dehydrogenase activity improves cardiac outcomes in a murine model of cardiac arrest

**DOI:** 10.1371/journal.pone.0185046

**Published:** 2017-09-21

**Authors:** Lin Piao, Yong-Hu Fang, Manfred M. Kubler, Michael W. Donnino, Willard W. Sharp

**Affiliations:** 1 Section of Emergency Medicine; Department of Medicine, University of Chicago, Chicago, Illinois, United States of America; 2 Departments of Emergency Medicine and Internal Medicine, Beth Israel Deaconess Medical Center, Boston, Massachusetts, United States of America; University of Otago, NEW ZEALAND

## Abstract

**Rationale:**

Post-ischemic changes in cellular metabolism alter myocardial and neurological function. Pyruvate dehydrogenase (PDH), the limiting step in mitochondrial glucose oxidation, is inhibited by increased expression of PDH kinase (PDK) during ischemia/reperfusion injury. This results in decreased utilization of glucose to generate cellular ATP. Post-cardiac arrest (CA) hypothermia improves outcomes and alters metabolism, but its influence on PDH and PDK activity following CA are unknown. We hypothesized that therapeutic hypothermia (TH) following CA is associated with the inhibition of PDK activity and increased PDH activity. We further hypothesized that an inhibitor of PDK activity, dichloroacetate (DCA), would improve PDH activity and post-CA outcomes.

**Methods and results:**

Anesthetized and ventilated adult female C57BL/6 wild-type mice underwent a 12-minute KCl-induced CA followed by cardiopulmonary resuscitation. Compared to normothermic (37°C) CA controls, administering TH (30°C) improved overall survival (72-hour survival rate: 62.5% vs. 28.6%, *P*<0.001), post-resuscitation myocardial function (ejection fraction: 50.9±3.1% vs. 27.2±2.0%, *P*<0.001; aorta systolic pressure: 132.7±7.3 vs. 72.3±3.0 mmHg, *P*<0.001), and neurological scores at 72-hour post CA (9.5±1.3 vs. 5.4±1.3, *P*<0.05). In both heart and brain, CA increased lactate concentrations (1.9-fold and 3.1-fold increase, respectively, *P*<0.01), decreased PDH enzyme activity (24% and 50% reduction, respectively, *P*<0.01), and increased PDK protein expressions (1.2-fold and 1.9-fold, respectively, *P*<0.01). In contrast, post-CA treatment with TH normalized lactate concentrations (*P*<0.01 and *P*<0.05) and PDK expressions (*P*<0.001 and *P*<0.05), while increasing PDH activity (*P*<0.01 and *P*<0.01) in both the heart and brain. Additionally, treatment with DCA (0.2 mg/g body weight) 30 min prior to CA improved both myocardial hemodynamics 2 hours post-CA (aortic systolic pressure: 123±3 vs. 96±4 mmHg, *P*<0.001) and 72-hour survival rates (50% vs. 19%, *P*<0.05) in normothermic animals.

**Conclusions:**

Enhanced PDH activity in the setting of TH or DCA administration is associated with improved post-CA resuscitation outcomes. PDH is a promising therapeutic target for improving post-CA outcomes.

## Introduction

Resuscitation following cardiac arrest (CA) causes post-ischemic cellular changes that alter myocardial and neurological function resulting in poor patient outcomes [1]. Therapeutic hypothermia (TH, 32–34°C) has been shown to improve post-CA resuscitation outcomes in animal studies and in clinical trials but, the mechanisms underlying the protective effects of TH are not fully understood [2, 3]. Previously, we have shown that myocardial ischemia following CA activates the mitochondrial fission factor, Drp1, resulting in excessive mitochondrial fission and myocardial dysfunction. We also found Drp1 inhibition, either pharmacologically or through TH, improved post-CA outcomes [4]. However, pharmacological inhibition of Drp1 was not as effective as TH in improving post-CA outcomes, demonstrating that TH likely has protective effects ancillary to its effects on mitochondrial fission.

The ATP required to maintain contractile function in heart and neurological function in brain is predominantly generated by glucose oxidation (GO) and fatty acid oxidation (FAO) in mitochondria [5]. Under pathophysiological conditions such as ischemia-reperfusion (IR), reduced rates of GO impairs myocardium contractility [5, 6]. Depressed GO has also been associated with impaired cerebral metabolism following ischemic injury and in neurodegenerative disorders such Alzheimer’s disease [7, 8]. Pyruvate dehydrogenase (PDH) is the rate-limiting enzyme that catalyzes the oxidative decarboxylation of pyruvate to form acetyl CoA, nicotinamide adenine dinucleotide (NADH) and CO_2_. PDH controls the flux of pyruvate into the mitochodria to initiate oxidative metabolism and is a key regulator of the citric acid cycle [9, 10]. Studies have shown that pyruvate dehydrogenase kinase (PDK) inhibits PDH by phosphorylating PDH during IR injury, thereby limiting the contribution of GO to the tricarboxylic acid cycle [6, 10–12].

Post-CA TH improves resuscitation outcomes and has known effects on cellular ATP utilization during ischemia [4], but its influence on GO in this setting is unknown. Here, we hypothesized that PDH activity is impaired following CA and that improved outcomes with TH are associated with improved PDH activity and the inhibition of PDK. We further examined whether dichloroacetate (DCA), a known activator of PDH activity, can improve post-CA resuscitation outcomes.

## Methods and materials

### Animal preparation, mice cardiac arrest surgery and survival measurement

In this study, we used a modified form of a previously published murine model of asystolic CA [3, 4]. The Institutional Animal Care and Use Committee of the University of Chicago, in accordance with National Institutes of Health guidelines, approved all animal procedures (see S1 File). Briefly, Female C57BL/6 mice (age, 6–8 months; 20–30 g) were anesthetized with 3% isoflurane and vascular access was acquired. Temperature, respirations, and EKG tracings were recorded continuously on a PowerLab data acquisition module (AD Instruments, Colorado Springs, CO). A 0.4 mm OD heparinized micro PE cannula (BioTime Inc., Berkeley, CA) was placed in the left jugular vein for fluid administration and right carotid artery for aortic systolic pressure (ASP) measurement. Asystolic CA was induced with a single bolus of KCl (0.8 mg/g) into the internal jugular vein and ventilation was suspended. Following 12 min of cardiac arrest, cardiopulmonary resuscitation (CPR) was performed at approximately 300–350 beats/min. After 90 seconds of CPR, 1.5 μg of epinephrine was injected followed by a saline flush. CPR was terminated when return of spontaneous circulation (ROSC), defined by a mean arterial pressure >40 mm Hg lasting longer than 5 minutes, was achieved. CPR was terminated if ROSC was not achieved after 5 minutes. Quality of CPR was retrospectively evaluated for each animal by reviewing CPR rates and arterial pressures. Resuscitated animals received IV 0.9% saline at a rate of 100 μL/hr and were monitored on mechanical ventilation for 120 minutes. Following CA, all of the resuscitated mice were unresponsive and did not require further anesthesia while intubated. Thereafter, they were extubated and allowed free access to food and water. There were no differences in time to extubation between treatment groups. Animals used for survival studies were returned to the animal facility and observed for 72-hour post-CA. The mice were observed every 2 hours during the first 6 hours following CA. In experiments requiring therapeutic hypothermia (TH), TH (within 2 minutes to reach 30°C) was induced at the start point of CPR as previously described [3]. Briefly, a latex glove filled with ice-water was applied to the mouse’s ventral surface immediately following the initiation of cardiac arrest to reach and maintain a rectal temperature of 30°C. In separate experiments, DCA (0.2mg/g body weight) or a PBS vehicle control, were administered 30 minutes prior to CA through an intraperitoneal injection in a blinded fashion. The samples for PDH activity, western blot, and immunostaining were obtained from the heart and brain tissues 15 minutes resuscitation after 12 min of CA.

### Mouse echocardiography

As previously described [4] two-dimensional M-mode echocardiography was performed 2-hours post-CA on mice anesthetized with 3% vaporized isoflurane. Mice were secured to a Vevo 2100 (VisualSonics, Toronto, ON, Canada) platform and monitored for temperature, heart rate, and electrocardiogram. Transthoracic echocardiography was performed using a parasternal long-axis approach to obtain 2D left ventricular images. M-mode images were used to measure left ventricular end-diastolic and end-systolic size, and to calculate the percent fractional shortening (FS%).

### Neurological scoring of animals

Neurological deficits post-CA (2-hour, 4 hour, 6-hour, 24-hour, 48-hour and 72-hour) were determined by a 12-point neurological scoring system used in mice [13]. Briefly, the neurological response was scored from 0 (no response or worst) to 2 (normal) for six responses: paw pinch, righting reflex, breathing, spontaneous movement, motor-global and motor-focal. Scoring was performed by a blinded observer. The scores for the six responses were added together to determine a total neurological score.

### PDH activity

As previously described [14], PDH activity was assessed with an anti-PDH antibody immobilized on a dipstick activity assay and was performed by following the manufacturer’s instructions (Abcam, Cambridge, MA). The colored precipitate will be quantified using Image J software (NIH, Bethesda, MD).

### Immunoblot

Protein samples from heart and brain tissues were isolated following the manufacturer’s instructions (Thermo Scientific, Rockford, IL). Western blots were performed using standard procedures as we described previously [15, 16]. Anti-PDH E1-alpha subunit (phosphor S293) (1:500) and anti-PDH E1-alpha subunit (1:500) were purchased from Abcam (Cambridge, MA, USA). Anti-PDK2 (1:1000), anti-PDK4 (1:1000) antibodies and anti-α-tubulin (1:2000) antibody are from Santa Cruz Biotechnology (Dallas, TX, USA). The secondary antibodies were from Cell Signaling Technology (Danvers, MA, USA).

### Immunofluorescence

Frozen sections were fixed in methanol, blocked with albumin (Sigma, St. Louis, MO), and incubated with primary antibodies for 1 hour at 25°C (anti-dystrophin antibody, 1:400 dilution, Abcam, Cambridge, MA, USA; anti-PDK4 antibody 1:500 dilution). Immunostaining was performed using standard procedures as we described previously [14].

### Statistics

Data was presented as means ± sem. Statistical analyses were performed using Prism software (Graph Pad, La Jolla, CA, USA). Inter-group differences were assessed by t-test or one-way ANOVA with post hoc analysis using Tukey’s test, as appropriate. Characteristics of ROSC and non-ROSC post CA were compared by Chi-square. Survival outcomes were compared via Long-rank analysis. Neurological scores were represented as ordinal outcomes and modeled using a cumulative link mixed model. Statistical differences between the groups were confirmed using a separate Wilcoxon rank-sum test at each time point (see S2 File). Values of *P*<0.05 were considered statistically significant.

## Results

### TH improves post-CA resuscitation outcomes

**Fig 1:**
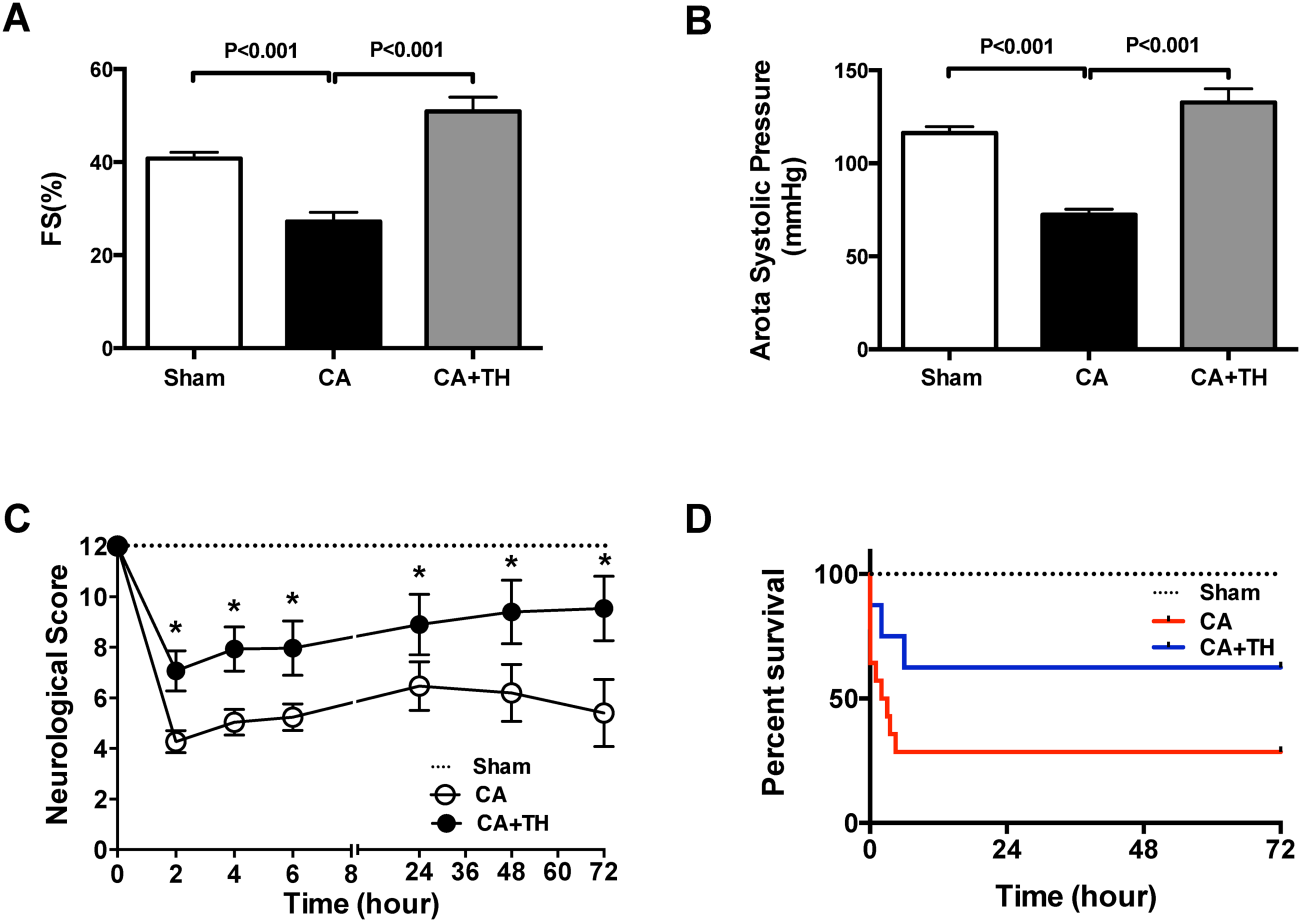
TH improves post-CA myocardial function, neurological outcomes and survival. Following twelve-minute asystolic CA, CPR was performed. Echocardiography showed fractional shortening (**A**, FS%) was reduced in the CA group and was improved by therapeutic hypothermia (TH). Aortic systolic pressure (**B**, ASP) was deceased following CA, but was elevated with TH. Neurological scores (**C**), and survival (**D**) were decreased following CA, but were improved with TH.

We first sought to confirm the effects of CA resuscitation on myocardial function with or without TH following a 12-minute CA. Fifteen minutes following ROSC, transthoracic echocardiography demonstrated decreased fractional shortening (FS %) compared to Sham operated mice (27.2±2.0% vs. 40.8±1.3%, *P*<0.001, n = 6–7, Fig 1A). Aortic systolic pressure (ASP) also was reduced in CA treated mice vs. Sham (72.3±3.0 vs. 116.3±3.4 mmHg, *P*<0.001, n = 6–7, Fig 1B). Mice treated with TH (30°C) demonstrated improved hemodynamics as evidence by an improved FS% to 50.9±3.1% (*P*<0.001 vs. CA group, n = 6, Fig 1A) and normalized ASP to 132.7±7.3 mmHg (*P*<0.001 vs. CA group, n = 6, Fig 1B). CA treated mice not only had decreased myocardial function, but also demonstrated worsened neurological scores at 72-hour post CA (5.4±1.3 vs. 12±0, *P*<0.001 vs. Sham, n = 15, Fig 1C) and decreased survival (Fig 1D). In contrast, TH improved neurological scores at all the time points according to separate Wilcoxon rank-sum tests (*P*<0.05, n = 15), especially at 72-hour post CA (9.5±1.3, *P*<0.05 vs. CA group, n = 15, Fig 1C). TH also improved the survival rate (62.5% vs. 28.6%, *P*<0.001, n = 24, Fig 1D).

### Metabolic changes in brain after CA

**Fig 2:**
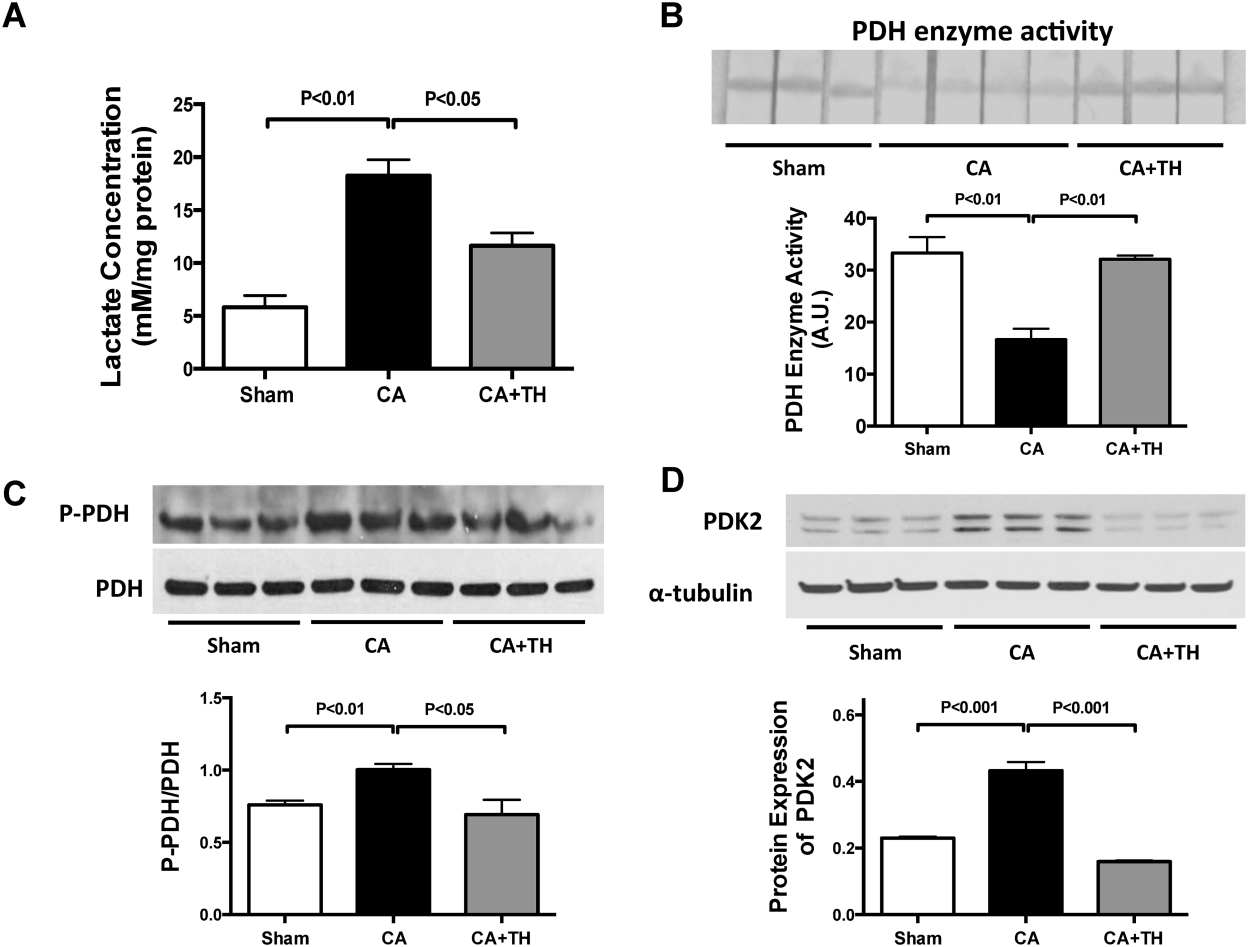
Post-CA inhibition of pyruvate dehydrogenase (PDH) in the brain is improved by TH. A. Brain lactate concentrations increased following CA, but not in mice treated with TH. **B.** PDH enzyme activity decreased following CA, whereas TH increased PDH enzyme activity as demonstrated by PDH dipstick activity assay. **C.** Phosphorylated PDH (P-PDH) increased following CA, but not in the TH group. **D.** Pyruvate dehydrogenase kinase (PDK) in the brain, PDK2, increased following CA, but not in mice treated with TH.

Lactate concentrations in the brain were markedly raised following CA resuscitation (3.1-fold increase, *P*<0.01, n = 3–4) in contrast to Sham and TH treated mice (*P*<0.05, n = 3, Fig 2A). In the brain, PDH enzyme activity was reduced 50% following CA (*P*<0.01 vs. Sham, n = 3–4), but was improved in CA animals treated with TH (*P*<0.01 vs. CA group, n = 3, Fig 2B). PDK2 is the major PDK isoform in the brain. Increased phosphorylation of PDH by PDK results in decreased PDH activity. Following CA, PDH exhibited increased phosphorylation (1.9-fold, *P*<0.01 vs. CA group, n = 3, Fig 2C) and PDK2 expressions increased 2.2-fold (*P*<0.01 vs. CA group, n = 3, Fig 2D). TH normalized phosphorylated-PDH and PDK2 expressions in the brain (*P*<0.01 and *P*<0.05, n = 3, respectively, Fig 2C & 2D). These results suggest that post-CA impairment of GO in the brain is mediated by PDK regulated reductions in PDH activity that can be reversed by TH. To determine if changes in glucose transporters in the brain could be affecting glucose oxidation, we examined the protein expressions of Glut3, the major glucose transporter in the brain, following CA. Neither CA nor TH changed Glut3 expression suggesting that glucose transport in the brain following CA is not altered. (*P*>0.05, respectively, n = 3, S1 Fig).

### Metabolic changes in heart after CA

**Fig 3:**
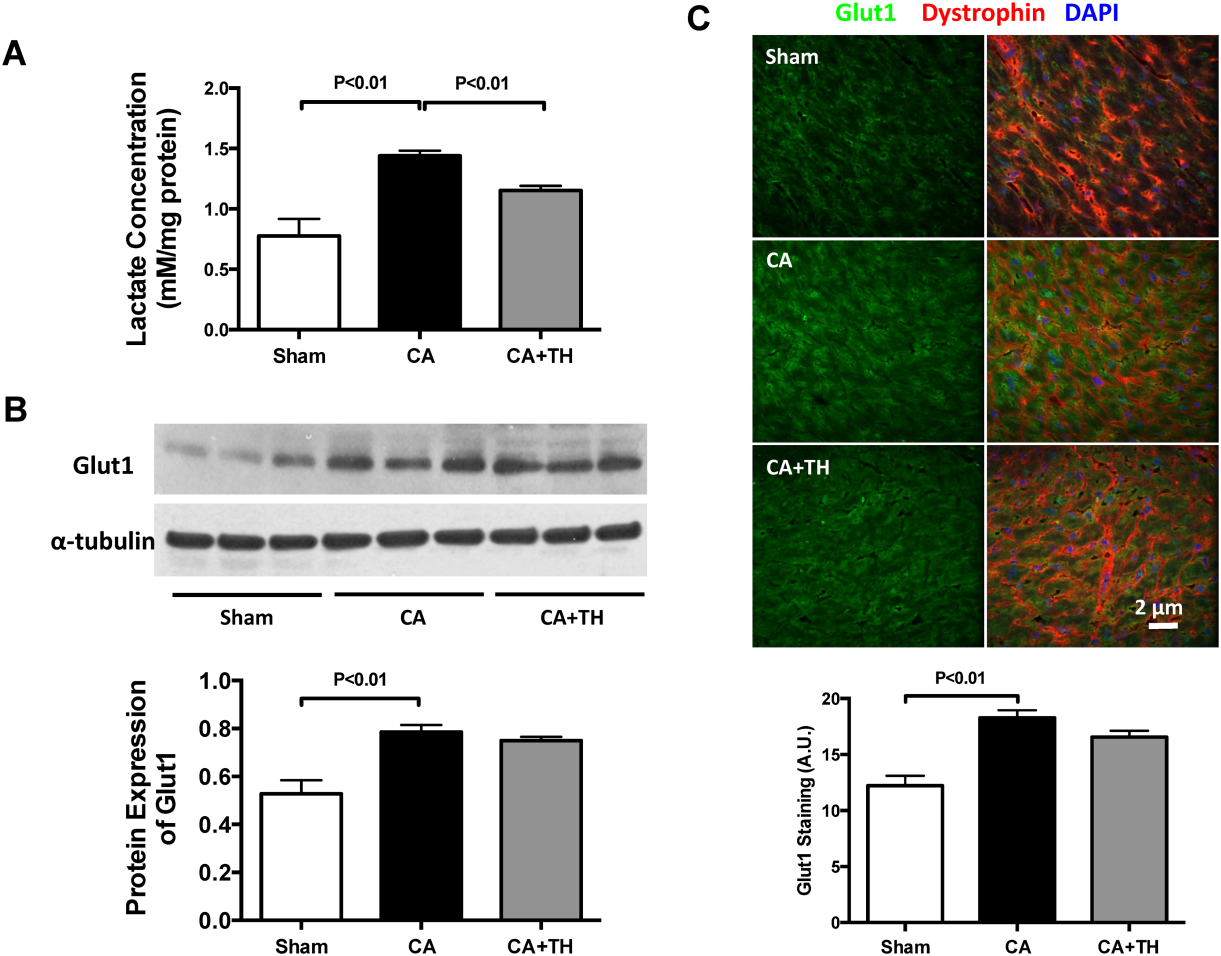
Lactate increases despite elevations in Glut1 expression in the post-CA myocardium. **A.** Lactate concentrations in the heart were elevated following CA, but normalized with TH treatment. **B&C.** Western blot and immunostaining showed that Glut1 was increased following CA, but not affected by TH.

**Fig 4:**
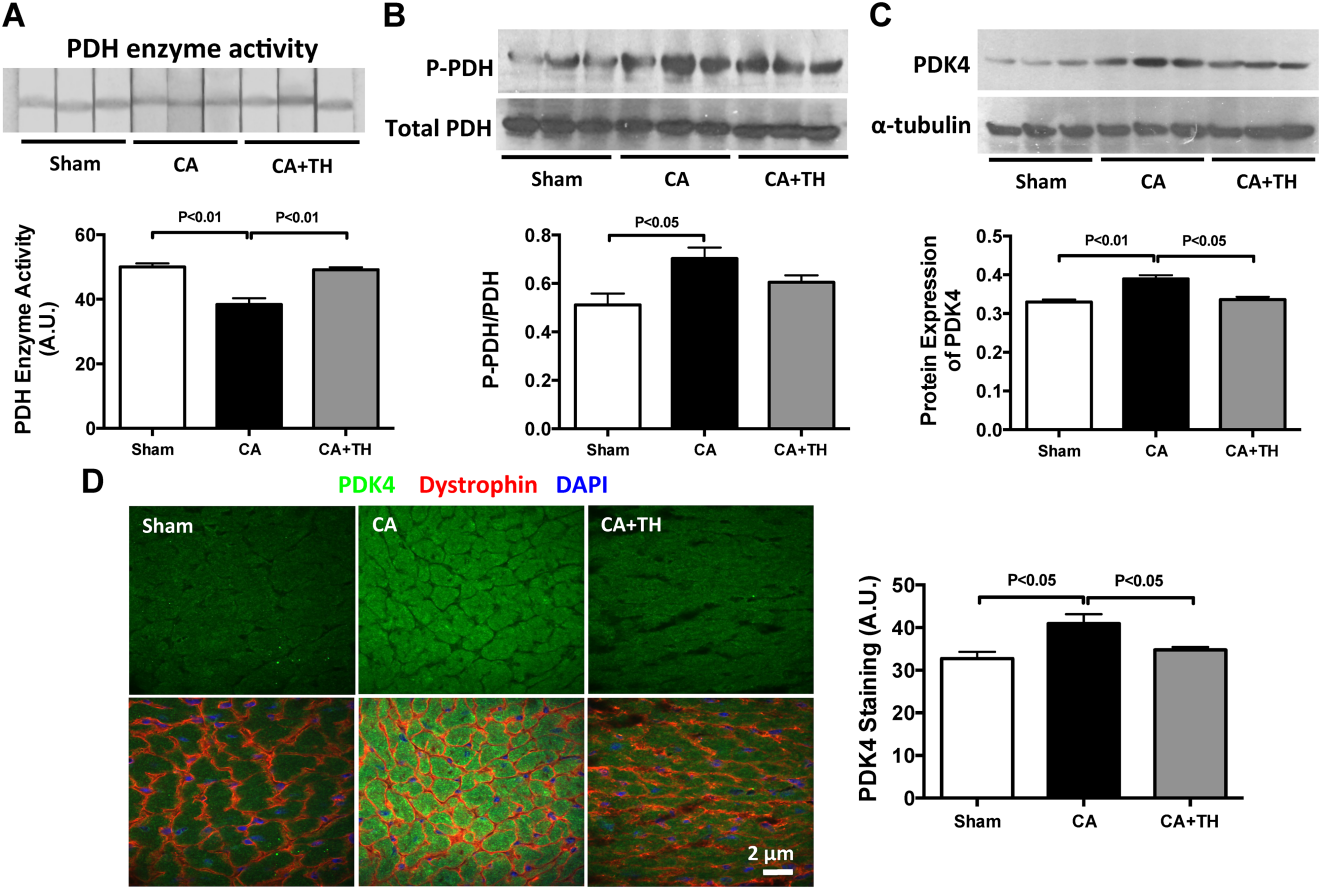
Post-CA inhibition of PDH in heart is improved by TH. **A.** PDH enzyme activity in the heart was decreased following CA, but was improved with TH. **B.** P-PDH in the heart was elevated following CA but was normalized with TH. **C&D.** PDK4, the primary PDK isoform in the heart, was elevated following CA but not in TH treated animals.

Lactate concentrations in the myocardium post-CA were increased (1.2-fold, *P*<0.01, n = 3–4, Fig 3A), and treatment with TH post-CA reduced this increase (*P*<0.01 vs. CA, n = 3, Fig 3A). To determine if alterations in glucose transporter expression is a potential contributor to increased myocardial lactate, we examined the protein expression of Glut1, the major glucose transporter in the heart, following CA with or without TH. CA increased Glut1 in the heart (*P*<0.01, respectively, n = 3–5, Fig 3B & 3C), but CA with hypothermia (TH) did not alter the expression of Glut1 (*P*>0.05, n = 3–5, Fig 3B & 3C). However, despite increased expression of Glut1 in the myocardium following CA, PDH enzyme activity was decreased in CA group (*P*<0.01 vs. Sham, n = 3, Fig 4A). The inhibition of PDH enzyme activity was further demonstrated by increased PDH phosphorylation following CA (1.3-fold (*P*<0.01 vs. Sham, n = 3, Fig 4B). In contrast, TH increased PDH activity (P<0.01, n = 3, Fig 4A) and decreased PDH phosphorylation in the heart (*P*<0.01, n = 3, Fig 4B). PDK4, the major inhibitory regulator of PDH was found to have increased 1.9 fold following CA (*P*<0.01 vs. CA group, n = 3, Fig 4C). Immunostaining of the myocardium also demonstrated a 1.2-fold increase of PDK4 protein in CA Group (*P*<0.05 vs. Sham group, n = 5, Fig 4D). Post-CA TH reversed the expressions of PDK4 as demonstrated by both immunoblotting and immunostaining (*P*<0.05, respectively, n = 3, Fig 4C & 4D). These findings demonstrate that there is impaired GO in the heart following CA despite the increased expression of the glucose transporter Glu1 and that TH is associated with improved PDH activity and decreased lactate in the heart.

### Dichloroacetate increases post-CA PDH activity

**Fig 5:**
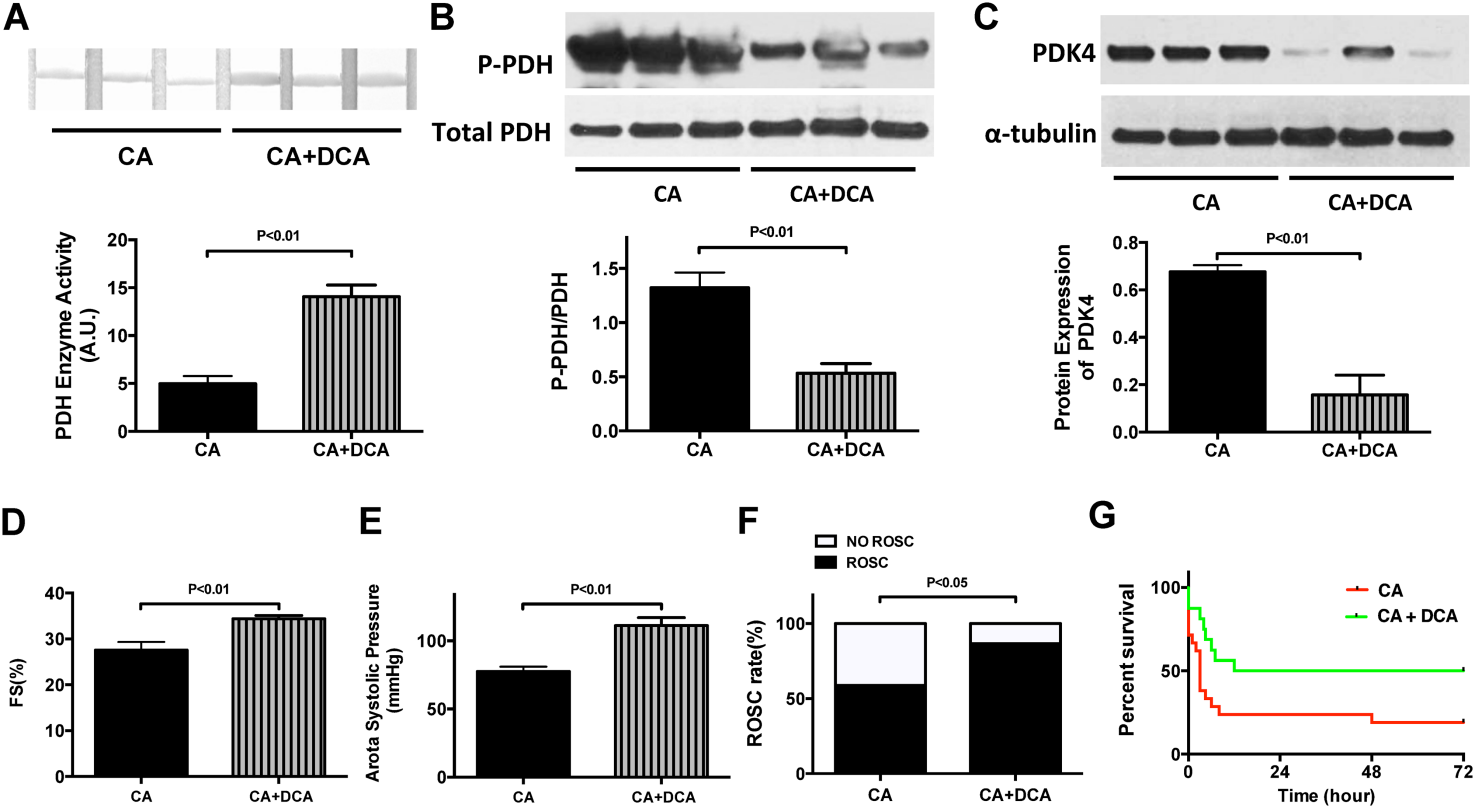
Dichloroacetate (DCA) treatment improves post-CA myocardial function and survival. **A**. PDH enzyme activity in the heart post-CA was improved by DCA administration. **B&C.** P-PDH and PDK4 in the heart post CA were both reduced by DCA treatment. **D&E.** FS% and ASP post-CA were both improved by DCA administration. **F&G.** ROSC rate and survival rate post-CA were both improved by DCA.

We next sought to determine if enhancing PDH activity by DCA, a well-known PDK inhibitor [17, 18], could improve post-CA outcomes. Pre-treatment with DCA 30 minutes prior to CA increased post-CA PDH activity in the heart as indicated by PDH activity assays and reduced phosphorylated-PDH westerns (*P*<0.01 vs. CA group, n = 3, respectively, Fig 5A & 5B). DCA treatment also decreased PDK4 expressions (*P*<0.01 vs. CA group, n = 3. Fig 5C), consistent with the enhancement of post-CA PDH activity. DCA also improved myocardial function (*P*<0.01 vs. CA group, n = 7, Fig 5D), hemodynamics (ASP, *P*<0.001 vs. CA group, n = 7–14, Fig 5E), and ROSC rate (*P*<0.05, n = 15–34, Fig 5F), and improved survival (50% vs. 19%, n = 24–28, *P*<0.05, Fig 5G). Collectively, these findings indicate that DCA enhances PDH activity in the heart and improves both myocardial function and survival outcomes post-CA. Despite these effects in the heart, pre-treatment with DCA did not change PDH activity in the brain and did not improve neurological outcomes at 72-hour post CA (*P*>0.05, n = 15 and n = 14, respectively, S2 Fig).

## Discussion

**Fig 6:**
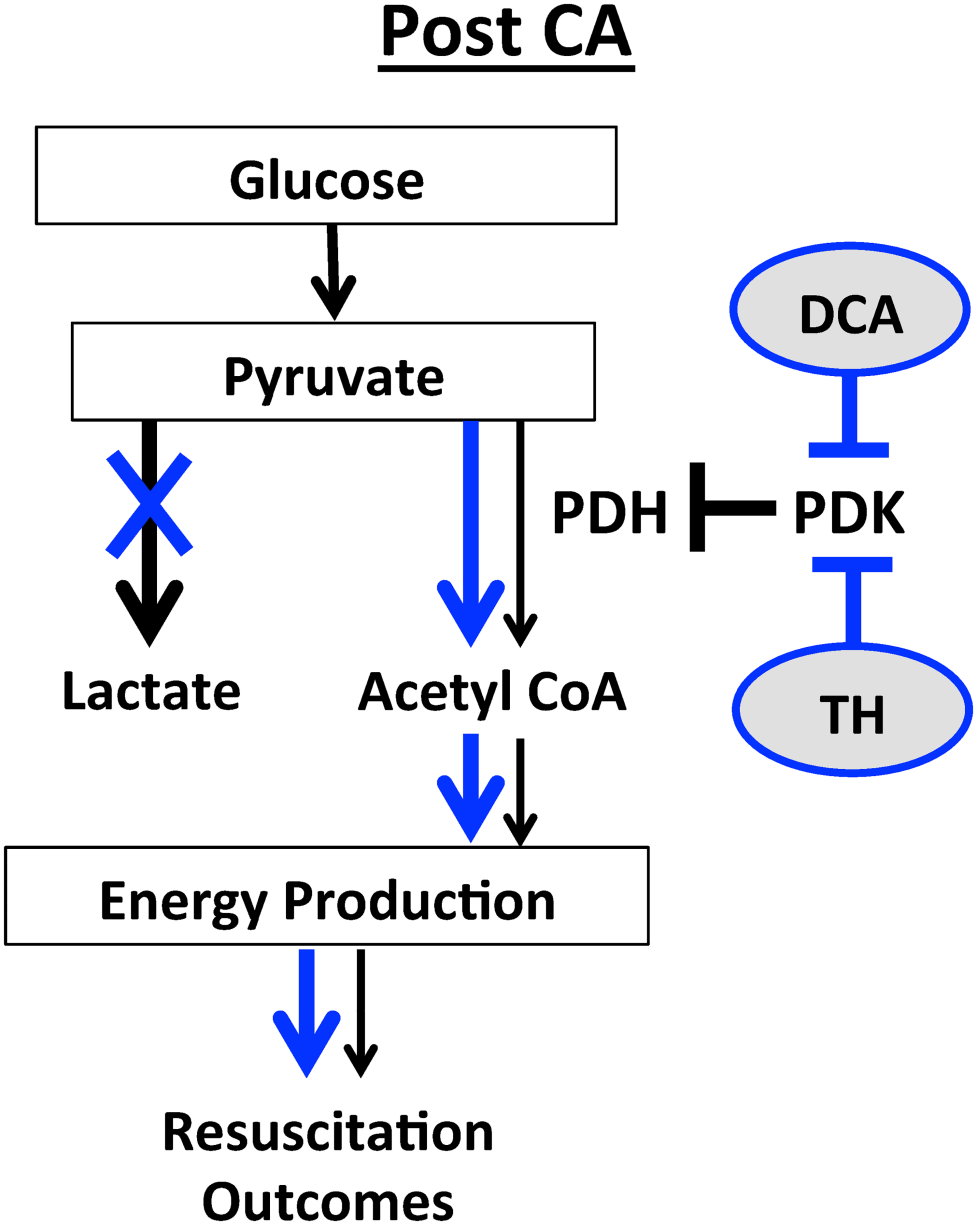
Metabolic alterations in PDH activity signaling in the heart and brain post-CA. There are metabolic imbalances in both brain and heart post-CA. GO is depressed evidence by decreased pyruvate, whereas glycosis is increased evidenced by increased lactate concentration. These are direct effects of PDK activation and PDH inhibition, which result in further cardiac and neurological dysfunction. Both TH and DCA, a PDK inhibitor, improve post-CA resuscitation outcomes, including cardiac output, neurological scores and survival by decreasing lactate concentrations, reducing PDK expressions, and elevating PDH activity.

This study has 3 main findings relevant to post-CA resuscitation: **1)** In both the heart and brain, there is a metabolic shift from GO to glycolysis following CA. This is evidenced by decreased PDH activity, increased PDK expression, and increased tissue lactate concentrations. These findings are associated with decreased cardiac function, increased neurological injury and depressed survival rates (Figs 1–4) **2**) Therapeutic hypothermia improves cardiac and neurological function, resulting in improved survival following CA. These improved outcomes are associated with improved PDH activity, reduced PDK expression, and decreased tissue lactate (Figs 1–4) 3) In the heart, PDH stimulation with DCA improves post-CA resuscitation outcomes including ROSC rate, myocardial function, and survival (Fig 5). Together, these findings suggest that PDH activity is inhibited following CA resuscitation resulting in the impairment of GO and a continued reliance on cellular glycolysis for energy production (Fig 6). Furthermore, this study establishes PDH activity as a possible therapeutic target for improving post-CA outcomes.

Patients with CA develop a severe metabolic acidosis and their blood pH can drop to 6.9 [19]. In canine models of ventricular fibrillation (VF) induced CA, PDH activity remains normal following 10 minutes of global cerebral ischemia. After 30 minutes of reperfusion post CA, there is a 70% decrease in PDH activity and it remains significantly depressed even after 24h [20] suggesting that decreased metabolic flux is responsible for elevations in lactate following CA [20, 21]. Together, these studies suggest there is a post-ischemic shift in metabolism from GO to anaerobic lactate generation during CA and that these metabolic changes persist following CA resuscitation. The findings of our study are congruent with these previous findings. Fifteen minutes following resuscitation from a 12-minute CA, Glut1 transporters are increased in the myocardium, suggesting increased glucose uptake. Despite these changes, PDH activity remains depressed and lactate concentrations remain elevated indicating a continued reliance of the myocardium on glycolysis for energy production. Continued post resuscitation reliance on glycolysis rather than GO in the brain is also evidenced by increased lactate, phosphorylated PDH and PDK2, and decreased PDH enzyme activity. In the heart, this metabolic switch from GO to glycolysis has been associated with myocardial infarction and congestive heart failure, resulting in decreased cardiac efficiency, and impaired heart function, and poor outcomes. Our findings extend these observations to asystolic CA (Figs 1–4). A unique aspect of this study is the dual examination of changes in metabolism of both the heart and brain following post-CA resuscitation. Compared to the heart, the brain demonstrated increased lactate (increase: 3.1-fold vs. 1.9-fold) and PDK expression (increase: 1.9-fold vs. 1.2-fold), as well as decreased PDH enzyme activity (decrease: 50% vs. 24%) following CA resuscitation (Figs 2–4). Since the brain is more dependent on glucose as an energy substrate than the heart and responds faster to changes in its oxygen and blood supply, these variations could be related to inherent metabolic differences between the brain and the heart [22].

DCA is a potent inhibitor of PDK [11, 17, 23] and therefore prevents transition of PDH from its active forms to the inactive phosphorylated form. As a result, PDH remains in an active form to promote the oxidation of glucose [17]. In published isolated Langendorff heart experiments, DCA pre-treatment significantly reduced lactate production and PDH phosphorylation following induction of ventricular fibrillation [24]. In a canine asphyxia CA model, DCA administration following CA reduced arterial and venous lactate concentrations. However, cardiac function, neurological outcomes, and survival were not assessed in this study [25]. Our present work demonstrates that DCA decreases the expressions of PDK4 and phosphorylated PDH. These DCA-induced changes are associated with increased PDH enzyme activity. Pre-treatment with DCA results in improved cardiac function evidenced by elevated FS% and ASP, both of which were associated with improved ROSC and survival (Fig 5). Although our findings demonstrate a relationship between DCA treatment and PDK expression in the heart, they do not determine whether this effect is directly or indirectly mediated by DCA. Decreased PDK expression following resuscitation may simply be the effect of improved cardiac hemodynamics following DCA therapy, rather than a direct effect of DCA on PDK expression. In contrast to the heart, we found that DCA had no effect on PDH enzyme activity in the brain and did not improve post-CA neurological scores (S2 Fig). Although one study reported that DCA elevates PDH activity and reduces brain injury by steadily crossing the blood brain barrier after hypoxia, it used an alternative, multi DCA dosing regimen making it difficult to compare to our study [23]. Further studies investigating the dosage and the delivery method of DCA are needed.

Moderate TH (34°C) following CA has beneficial effects on neurologic recovery [26], cerebral function [27], and morphologic outcomes [28] in dogs. In an asphyxia-induced murine model of CA, TH improves cardiac output, hemodynamic parameters and survival rate [29]. TH (32°C -34°C) is also used as a neuroprotective treatment in CA patients following the outcomes of two clinical trials. It improves neurological outcomes and decreases mortality [30–32]. TH also has beneficial effects on left ventricular ejection fraction and the improved cardiac function is associated with an improved survival over time [33]. Nevertheless, studies investigating the mechanisms underlying the protective effects of TH post-CA are limited. In rats following global ischemia, TH demonstrated no direct effect on local cerebral blood flow or free fatty acid accumulation, but completely inhibited glutamate release and significant reduced dopamine release [34]. In vitro and ex vivo studies on the protective effects of TH in IR injury show that hypothermia stabilizes mitochondrial membrane potential, reduces apoptosis and preserves cellular energy production [35, 36]. These effects may be mediated in part by reducing mitochondrial permeability transition pore (MPTP) opening, thus protecting mitochondria from IR injury [29]. In one study using isolated hearts, ^13^C NMR spectra showed that severe hypothermia, ranging from 32°C to 17°C, gradually depressed the production of fatty acids and increased the production of pyruvate or lactate [37] suggesting that the heart begins to use GO instead of FAO in the setting of hypothermia. This may be because GO is a more efficient energy path than fatty acid oxidation [37, 38]. Simple stoichiometry (by calculating phosphorylation/oxygen reuptake ratio) suggests that fatty acid oxidation approximately costs 12% more oxygen than GO to generate a given amount of ATP. Also, ventricular mechanical efficiency (measured as left ventricular pressure volume area related to myocardial oxygen consumption) is reduced if glucose oxidation is switched to fatty acid oxidation [39]. Fatty acids increase voltage-gated calcium channel activity and Na^+^/Ca^2+^ exchange. Thus, the “oxygen-wasting” effect of fatty acid oxidation may be linked to calcium handling in the heart [39]. In our study, post-CA TH was associated with increased PDH activity, improved cardiac and neurological function, and enhanced survival. These findings demonstrate that TH not only preserves energy use, but also plays an important role in regulating post ischemic metabolic pathways and moderating mitochondrial functions.

### Limitations

This study has several important limitations. The first is that we used a model of asystolic CA and thus some of our findings may not be generalizable to all forms of CA. Secondly, we used a murine model recognizing that findings from small animals may not completely reflect larger animal physiology. Thirdly, we have not performed direct metabolic labeling studies to measure glucose or free fatty acid oxidation in the post-CA setting [34]. Futures studies are needed to directly assess the role of glucose and fatty acid oxidation in the post-CA setting with or without TH. Such studies could give greater insight into whether PDH activity is directly stimulated by TH. Finally, in this study, DCA was administered prior to CA. Our intention was to demonstrate proof of principle that changes in PDH and PDK activity could be protective in the CA setting. Future studies will be needed to elucidate whether post-CA manipulation of PDH activity can improve post-CA outcomes either by DCA or by the administration of other known PDK inhibitors.

## Conclusions

Post-CA resuscitation results in metabolic imbalances in both the heart and brain which contribute to poor resuscitation outcomes. Following resuscitation, mitochondrial GO in both the heart and brain is depressed resulting in increased cellular glycolysis and lactate accumulation. TH reverses these metabolic imbalances, improves PDH activity, and improves post-CA resuscitation outcomes suggesting PDH as a promising potential therapeutic target. Although, the PDK inhibitor DCA improved PDH activity in the heart and was associated with improved cardiovascular function, it did not impact PDH activity in the brain and was not associated with improved neurological outcomes. Collectively, these findings indicate that PDH activity is a promising therapeutic target for improving post-CA resuscitation.

## Supporting information

S1 Fig Glucose transporter Glut3 is not altered in the brain post-CA. The western blot bands and mean data showed no changes of Glut3 expression in the brain post CA.
(TIFF)

S2 Fig DCA administration did not improve post-CA neurological outcomes. DCA administration (0.2mg/g body weight) 30 minutes before CA had no significant effect on PDH enzyme activity (**A**) and on post-CA neurological scores (**B)**.
(TIFF)

S1 File Animal Care and use. (DOCX)

S2 File Neurological score analysis. (DOCX)

S1 Dataset (PZFX)
