# Supplementary figures and images for: Enhanced pyruvate dehydrogenase activity improves cardiac outcomes in a murine model of cardiac arrest
Source: PLoS One. 2017 Sep 21;12(9):e0185046. doi: 10.1371/journal.pone.0185046 (PMC5608301; doi:10.1371/journal.pone.0185046)

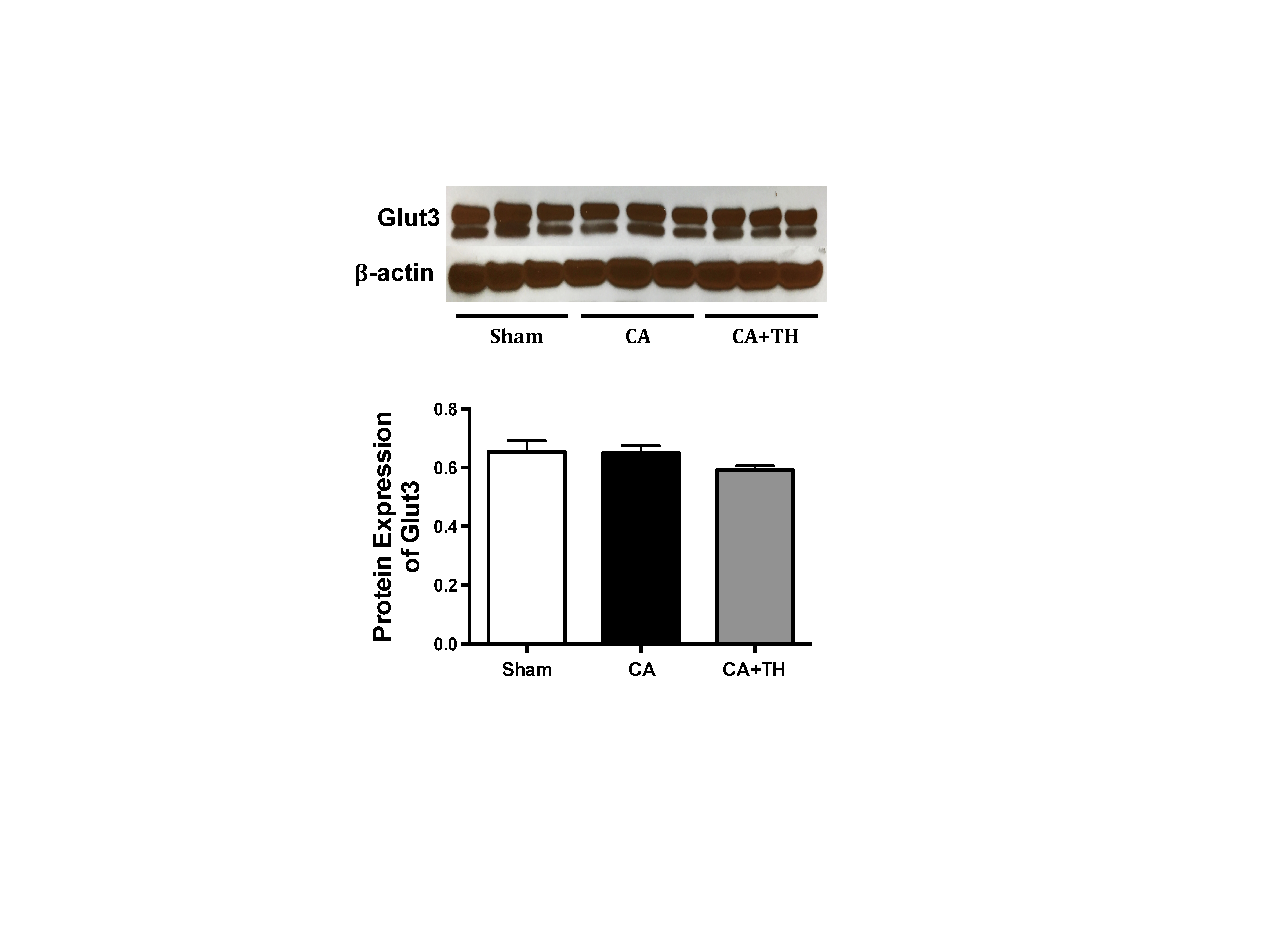

Supplement: S1 Fig — The western blot bands and mean data showed no changes of Glut3 expression in the brain post CA. (TIFF) [file pone.0185046.s001.tiff]

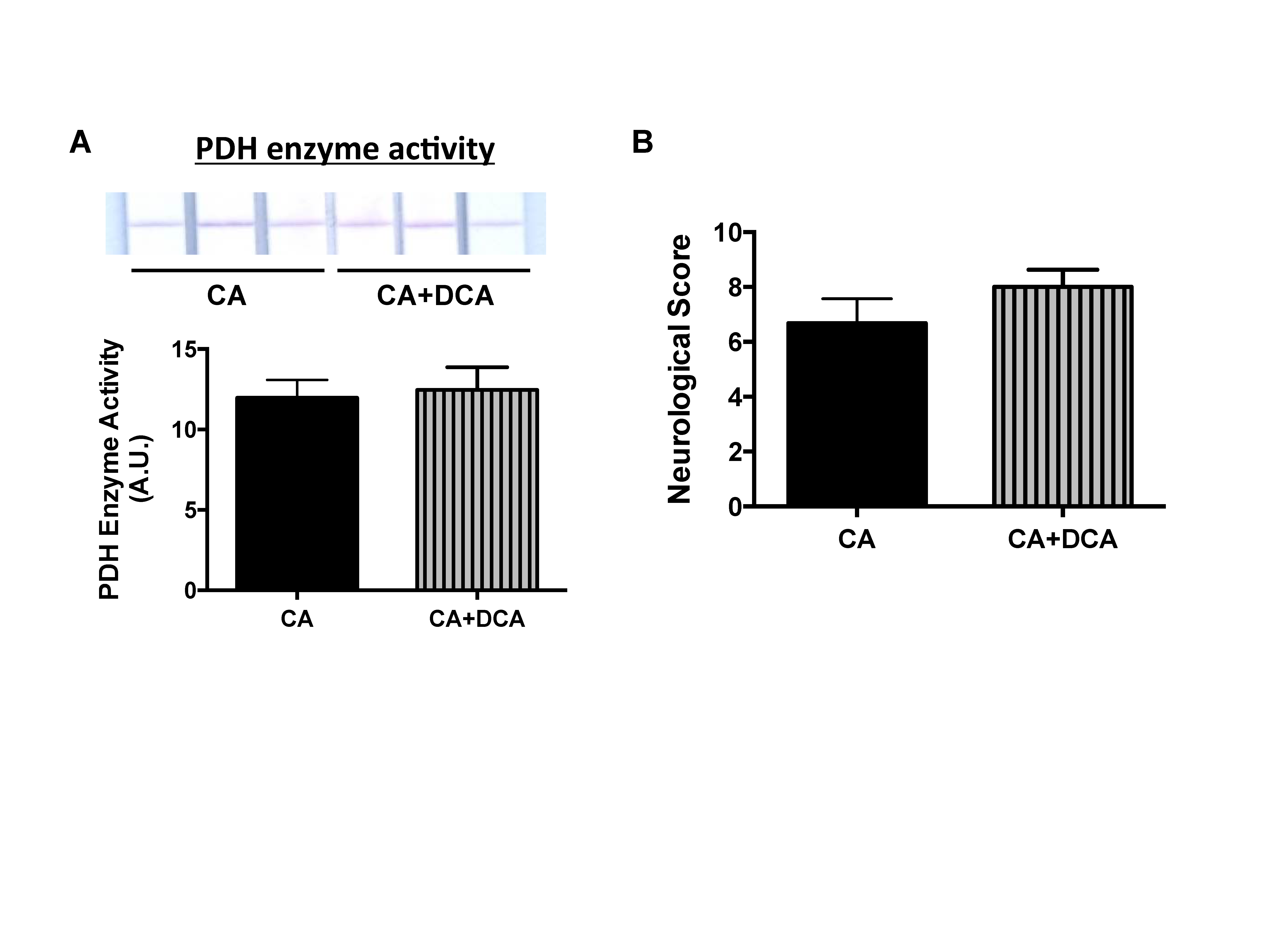

Supplement: S2 Fig — DCA administration (0.2mg/g body weight) 30 minutes before CA had no significant effect on PDH enzyme activity (A) and on post-CA neurological scores (B). (TIFF) [file pone.0185046.s002.tiff]
