# Supplementary material for: Enhanced pyruvate dehydrogenase activity improves cardiac outcomes in a murine model of cardiac arrest
Source: PLoS One. 2017 Sep 21;12(9):e0185046. doi: 10.1371/journal.pone.0185046 (PMC5608301; doi:10.1371/journal.pone.0185046)
Supplement: S1 File — (DOCX) [file pone.0185046.s003.docx]

**Supplemental Material**

**Animal Care and Use**

Survival study animals remain on mechanical ventilation and are given maintenance intravenous fluids for 2-3 hours until they show evidence of spontaneous breathing along with adequate mean arterial pressures (> 55 mmHg).  At this time, animals are disconnected from the ventilator for a breathing trial.  Once it is determined that the animal is able to breathe on its own, the animal is extubated and all vascular access devices are removed, vessels ligated and surgical wounds sutured as described elsewhere. If ketamine/xylazine is used for anesthesia, prior to surgical repair, animals will receive an additional intraperitoneal injection of anesthesia (ketamine/xylazine) as needed to ensure adequate sedation/pain control. Once extubated and de-instrumented, the animals are placed in a protected recovery area and visually monitored for 4-6 hours. A normal breathing pattern, and the ability of the mouse to maintain upright positioning and regulate body temperature are ensured before animals are returned to their cages in the animal facility for monitoring.  Once animals are able to maintain a normal breathing pattern and upright positioning, they are returned to an approved disposable cage and transferred to return facility for long-term survival monitoring.  Post arrest mice that do not attain these milestones within 6 hours of ROSC will be euthanized.

Following resuscitation, animals are generally neurologically devastated and do not generally exhibit evidence of pain within the first 6 of resuscitation.  Some animals die of neurologic or sudden cardiac sequela that we believe are not painful.  Post-op analgesics are not routinely used because they may alter inflammatory responses, which could influence the recovery and research objectives (Sabol Jones & Ross, 1995).  However, if animals do exhibit evidence of pain following resuscitation or during daily monitoring, post-op analgesics will be administered or the mouse will be removed from the protocol. Isoflurane administration followed by cervical dislocation was used to euthanize animals that met these criteria for euthanasia.

All animal procedures were approved by the University of Institutional Animal Card and Use Committee (IACUC).
