# Supplementary material for: Enhanced pyruvate dehydrogenase activity improves cardiac outcomes in a murine model of cardiac arrest
Source: PLoS One. 2017 Sep 21;12(9):e0185046. doi: 10.1371/journal.pone.0185046 (PMC5608301; doi:10.1371/journal.pone.0185046)
Supplement: S2 File — (DOCX) [file pone.0185046.s004.docx]

Overall summary table

----------------------------------------------------------------------------

| TIME

GROUP | 2 4 6 24 48 72

----------+-----------------------------------------------------------------

ca | 15 15 15 15 15 15 N

| 4.2666667 5.0333333 5.2333333 6.4666667 6.2 5.4 Mean

| 1.69944 1.986262 2.04299 3.710346 4.35808 5.151976 SD

| 5 5.5 5.5 8 8 7 Median

|

th | 15 15 15 15 15 15

| 7.0666667 7.9333333 7.9666667 8.9 9.4 9.5333333

| 3.075634 3.390463 4.163904 4.626013 4.877792 4.9406

| 8 9 10 11 12 12

|

Total | 30 30 30 30 30 30

| 5.6666667 6.4833333 6.6 7.6833333 7.8 7.4666667

| 2.826394 3.103067 3.509593 4.302131 4.827365 5.386659

| 5.75 6.75 6.75 9 10 11

----------------------------------------------------------------------------

Individual animal trajectories:

Mean +/- 1 SE plots:

Cumulative distribution plots showing differences in distribution of scores between groups at all timepoints:

In the CA group, none of the animals had returned to full normal function (score=12) at times 2-48, and only 13% had returned to full function at 72 hours.

In the TH group, none of the animals had returned to full normal function (score=12) at times 2-6, but 7% had returned to full function at 24 hours and 53% and 73% had full function by 48 and 72 hours, respectively.

Scores were categorized as 0-3.9=1, 4-5.9=2, 6-7.9=3, 8-9.9=4, 10-12=5 and ordinal logistic mixed effects models were fit (i.e., cumulative link mixed model).

Group = CA

RECODE of | TIME

score | 2 4 6 24 48 72 | Total

-----------+------------------------------------------------------------------+----------

1 | 4 2 2 4 5 7 | 24

| 26.67 13.33 13.33 26.67 33.33 46.67 | 26.67

-----------+------------------------------------------------------------------+----------

2 | 9 7 7 0 0 0 | 23

| 60.00 46.67 46.67 0.00 0.00 0.00 | 25.56

-----------+------------------------------------------------------------------+----------

3 | 2 5 4 3 1 1 | 16

| 13.33 33.33 26.67 20.00 6.67 6.67 | 17.78

-----------+------------------------------------------------------------------+----------

4 | 0 1 2 6 5 3 | 17

| 0.00 6.67 13.33 40.00 33.33 20.00 | 18.89

-----------+------------------------------------------------------------------+----------

5 | 0 0 0 2 4 4 | 10

| 0.00 0.00 0.00 13.33 26.67 26.67 | 11.11

-----------+------------------------------------------------------------------+----------

Total | 15 15 15 15 15 15 | 90

| 100.00 100.00 100.00 100.00 100.00 100.00 | 100.00

Group = TH

RECODE of | TIME

score | 2 4 6 24 48 72 | Total

-----------+------------------------------------------------------------------+----------

1 | 2 2 3 3 3 3 | 16

| 13.33 13.33 20.00 20.00 20.00 20.00 | 17.78

-----------+------------------------------------------------------------------+----------

3 | 4 1 0 0 0 0 | 5

| 26.67 6.67 0.00 0.00 0.00 0.00 | 5.56

-----------+------------------------------------------------------------------+----------

4 | 8 6 3 0 0 0 | 17

| 53.33 40.00 20.00 0.00 0.00 0.00 | 18.89

-----------+------------------------------------------------------------------+----------

5 | 1 6 9 12 12 12 | 52

| 6.67 40.00 60.00 80.00 80.00 80.00 | 57.78

-----------+------------------------------------------------------------------+----------

Total | 15 15 15 15 15 15 | 90

| 100.00 100.00 100.00 100.00 100.00 100.00 | 100.00

There was a significant overall difference between ca and th groups (p=0.01) which did not appear to vary significantly by time (time group interaction p= 0.73 from 5-df test and p=0.21 from 1-df test). This was confirmed using separate Wilcoxon rank-sum tests at each timepoint.
